# Dental caries status of students from migrant primary schools in Shanghai Pudong New Area

**DOI:** 10.1186/s12903-016-0187-y

**Published:** 2016-03-05

**Authors:** Cheng-jun Liu, Wei Zhou, Xue-shan Feng

**Affiliations:** School of Public Health, Fudan University, NO.130 Dong-An Road, Xuhui, Shanghai, 200032 China; Key Laboratory of Public Health Safety (Fudan University), Ministry of Education, NO.130 Dong-An Road, Xuhui, Shanghai, 200032 China; Eye and Dental Diseases Prevention & Treatment Center of Pudong New Area, NO. 222 Wen-Hua Road, Pudong New Area, Shanghai, 201399 China

**Keywords:** Caries, Behaviours, Migrant children, China

## Abstract

**Background:**

In China, there is a large migrant population. A significant proportion of children of the migrant population in China are not able to attend public schools due to the lack of local household registration (HuKou). They turn to privately-operated migrant schools, which are usually under-funded, have bad environmental facilities and are inadequately staffed compared to public schools. This study aims to describe the dental caries status of students from migrant primary schools in Shanghai Pudong New Area and factors that influence their caries status.

**Methods:**

Children (7–12 years old) from migrant primary schools in Shanghai Pudong New Area were randomly selected through a multi-stage cluster sampling method. Following the recommendation of the World Health Organization, caries experiences were recorded using the dmft index. A questionnaire to survey the children’s socio-demographic characteristics and oral health-related behaviours was completed by the children’s parents or guardians.

**Results:**

A total of 1385 children in migrant primary schools were invited, of which 1323 joined the survey (95.5 %). Among all the surveyed subjects, the prevalence rate of dental caries was 74.7 % (65.7 % for primary teeth and 28.1 % for permanent teeth). The mean (SD) dmft scores were 3.17 (3.12), 2.74 (3.02) for the primary teeth and 0.44 (0.84) for the permanent teeth, and 99.5 % of the carious teeth received no treatment.

**Conclusions:**

Students from migrant primary schools in Shanghai Pudong New Area had bad conditions of dental caries and most of the carious teeth were left untreated. The caries experience was associated with tooth brushing habits, snacking habits, dental visit and gender.

## Background

In the past 20 years, the migrant population has become a global problem [1]. In China, the reform, opening up and urbanization drives a large number of people away from rural areas to cities [2]. The number of these people increased from 30 million in the 1980s to 236 million in 2010, accounting for more than 15 % of the total national population [3]. The majority of the migrant population in cities do not possess local household registration (HuKou), and are therefore excluded from full access to pension, health care, public education and other social benefits at the place where they live. Based on the 2010 Census, there are around 210 million such “non-HuKou” migrants who work and live in a city other than where his/her official HuKou is registered. Among all non-HuKou migrants, approximately 20 million are children aged between 6 and 14 [4].

After migrating to the city, there are two different types of school settings for migrant children: public schools and migrant children schools. Public schools are established by the government for the majority of local urban children and have qualified teachers, good environmental facilities and adequate funds [5]. In public schools, migrant children study and live together with local urban children [6]. However, without a local urban HuKou, migrant children can only temporarily enrol in public schools as transient students. Migrant children’s schools, on the contrary, are established specifically for migrant children by migrants themselves in response to the increasing demand for schooling among growing numbers of migrant children in the city. These schools are located in rural migrant communities, and the students are typically from families that have moved from rural areas in the various provinces of China [7]. However, these migrant children schools are usually under-funded, have bad environmental facilities and are inadequately staffed compared to public schools [5].

Shanghai is the economic centre of eastern China. It had a population of 24.15 million in 2013, 41 % of which is migrant population [8]. The data from the Shanghai Education Commission in 2012 showed that Shanghai had about 380,000 children of migrant population and 60 % of them went to public schools, leaving 150,000 to 155 migrant children schools. Among these schools, 95 % were primary schools, and they were mainly located in 9 districts and 1 county within Shanghai (Shanghai has 16 districts and 1 county altogether). Pudong New Area is the biggest district in Shanghai. By the end of 2013, Pudong New Area had a population of 5.45 million, 48 % of which was migrant population [9]. It has 59 migrant children primary schools, in which about 36,000 students are enrolled.

Despite the fact that China has already established a national oral health monitoring system and conducted a number of epidemiological researches, these studies excluded the migrant children due to their non-resident status (without a local urban HuKou) [10]. There is scarce information on prevalence and severity of dental caries, oral health-related behaviours of migrant children in Shanghai. With poor family background, students from migrant primary schools may have bad conditions of dental caries.

Using the students from migrant primary schools in Pudong New Area as the target population, this study aims to achieve a better understanding of the dental caries status and factors that influence the caries status of students from the underprivileged migrant population in Shanghai.

## Methods

### Selection of children and sample size

As Pudong New Area has a vast area (1210 km^2^) with great variation of economic development and population density, this study used a randomised multi-stage cluster sampling method to select the subjects. Firstly, the whole geographic area was divided into three stratifications (the developed area along the Huangpu River covering 14 sub-districts, the under-developed area along the sea covering 9 sub-districts, and the moderately developed area covering 13 sub-districts). Two migrant primary schools were chosen randomly from each stratification. Secondly, in each of the 6 selected schools, one class was sampled randomly from each grade. All children aged 7 to 12 in selected classes were invited to participate in this study. The study protocol was approved by the Ethics Committee of Pudong New Area Eye and Dental Disease Prevention and Treatment Centre. Children with written informed consent of their parents or guardians, and in good general health were included. Children with major systemic diseases or syndromes, or on long-term medication (four with mental retardation), representing 0.3 percent of the respondents, were excluded from the study.

According to the third national oral-health survey conducted in 2005, the prevalence of primary tooth caries of 5-year-old groups and permanent caries of 12-year-old groups in East China was 69.7 % and 33.3 %, respectively [10]. This study calculated the sample size with the estimation that the primary and permanent caries prevalence approximated to that in the larger area of East China. With a standard error set at 1.5 %, the sample size required in this survey would be 939 and 987, respectively. The response rate was expected to be 90 %, and as such, this study aimed to recruit 1097 children.

### Questionnaire

A self-administered questionnaire was completed by children’s parents or guardians before caries examination. The 6 participating schools’ teachers facilitated the distribution and collection of questionnaires. The questionnaire used in this study was designed according to the questionnaire used in the third national epidemiology stomatology sampling survey [10]. The questionnaire was structured to collect information on: (a) demographic background (age, gender, occupation and education level of parents or guardians, family income); (b) residence history of children and parents or guardians; (c) children’s oral health-related behaviours (tooth brushing practice, the child’s snacking habits and dental visits) and (d) parents’ or guardians’ knowledge and attitude of the child’s oral health.

### Caries examination

The surveyed children’s caries examinations were conducted by a trained dentist using a 0.5 mm ball-ended CPI probe and a disposable dental mirror in the migrant schools. Dental caries was accessed using criteria recommended by the WHO [11]. These students were not required to clean their teeth before caries examination. No radiography examination was performed on the students. To measure the internal consistence of dentists, 5 % of the subjects were selected randomly for repeated tests.

### Results feedback

After the caries examination, a report explaining the child’s dental caries status and proposing necessary treatment for dental caries was sent to the child’s parents or guardians.

### Data analysis

All analyses were performed using a statistical software package (IBM SPSS Statistics, version 20). Caries prevalence (% with primary and permanent caries) and mean dmft scores were calculated for the surveyed children. The socio-economic status of the subjects, children’s oral health-related behaviours, parents’ knowledge and attitude of the child’s oral health were derived from descriptive statistics. Comparisons were made using an independent *t*-test (2 categories) and one-way ANOVA (more than 2 categories) to assess the statistical significance of the differences in the dental caries experience (mean dmft scores). Multiple comparisons using the Bonferroni test were performed to compare the groups (N >2) where the independent variable was found to be a significant factor affecting the caries experience (mean dmft scores). The Chi-square test was used to compare proportions. Analysis of multiple linear regression was performed to investigate the effects of the independent variables studied on the child’s dental caries experience (dmft scores). The dependent variable was the child’s dmft scores. The independent variables were gender, parents’ educational background (not educated or primary, junior high or above), family income (annual income per person ≤ 10000 Yuan, annual income per person > 10000 Yuan), tooth brushing twice or more daily (yes, no), age of initial brushing (≤3, >3),sweet snacks before sleep without tooth brushing (often/occasionally, never), daily sweet snacks (yes, no), parents’ knowledge/attitudes of the child’s oral health(agree, disagree, unknown). Intra-examiner reliability was evaluated by Cohen’s Kappa statistics for dental caries examination. The statistical significance level for all tests was set at 5 %.

## Results

### Basic characteristics of subjects

A total of 1385 children from 6 schools were invited; 1323 attended the caries examination and questionnaire. The response rate was 95.5 % (1323/1385). Among those who did not attend the survey, 23 children were absent on the day of examination, and 39 did not submit written informed consent or the questionnaire. The average time for 1323 surveyed children to live in Shanghai was 5.07 ± 3.07 years. The socio-demographic characteristics of the surveyed children and their parents are presented in Table 1.

**Table 1:** Socio-demographic characteristics of the surveyed children and their parents

Characteristics	n	%
Age (years old)		
7–8	376	28.42
9–10	436	32.96
11–12	511	38.62
Gender		
Boy	745	56.31
Girl	578	43.69
Father’s educational background		
Not educated	34	2.57
Primary	1066	80.57
Junior high	193	14.59
Senior high and above	30	2.27
Mother’s educational background		
Not educated	86	6.50
Primary	1097	82.92
Junior high	117	8.84
Senior high and above	23	1.73
Father’s occupation		
Labour/manual worker	703	53.14
Self-employed	24	1.81
Business service people	343	25.93
Agricultural labour	60	4.54
Others	193	14.59
Mother’s occupation		
Labour/manual worker	598	45.20
Self-employed	9	0.68
Business service people	312	23.58
Agricultural labour	60	4.54
Others	344	26.00
Family income (annual income per person, Yuan)^a^		
Mean (SD)	14404(16632)	
Median	10000	
Total	1323	100.00

^a^Average family annual income of migrant population was lower than the annual disposable income which was 40188 Yuan of local urban residents and that of local rural residents which was 17401 Yuan respectively (Data source: Shanghai Statistics Bureau, 2012 Shanghai National Economy and Social Development Statistical Bulletin, www.http://www.stats-sh.gov.cn/sjfb/201302/253153.html)

### Caries status of surveyed children

The internal consistency in this examination was very high (Kappa = 0.94). The dental caries prevalence and severity of the surveyed students were summarized in Table 2. An overwhelming majority (74.7 %) of students were affected by caries (primary teeth 65.7 %; permanent teeth 27.7 %). The dmft of primary and permanent teeth were 2.74 and 0.44 respectively. Almost all (99.5 %) affected teeth were untreated decayed teeth.

**Table 2:** Dental caries prevalence and experience of surveyed children with different ages and genders

Variable	Group	n	% dmft > 0	dt	mt	ft	dmft(SD)
Primary teeth							
Age (years old)	7–8	376	81.4*	4.20	0.01	0.02	4.22 (3.57)**
	9–10	436	81.0*	3.29	0.02	0.02	3.33 (2.73)**
	11–12	511	41.1*	1.13	0.00	0.01	1.13 (1.83)**
Gender	Boy	745	67.0	2.69	0.01	0.01	2.72 (2.92)
	Girl	578	64.0	2.74	0.00	0.02	2.76 (3.14)
Total		1323	65.7	2.71	0.01	0.01	2.74 (3.02)
Permanent teeth							
Age (years old)	7–8	376	14.6***	0.22	0.00	0.00	0.22 (0.61)****
	9–10	436	32.1***	0.47	0.00	0.01	0.48 (0.83)****
	11–12	511	33.5***	0.56	0.00	0.00	0.56 (0.95)****
Gender	Boy	745	23.0*****	0.35	0.00	0.00	0.35 (0.76)******
	Girl	578	33.7*****	0.55	0.00	0.01	0.55 (0.91)******
Total		1323	27.7	0.43	0.00	0.00	0.44 (0.84)
Primary and permanent teeth	1323	74.7	3.15	0.01	0.00	3.17(3.12)	

*Significant difference in the three age groups (*P <* 0.001)

**Significant difference in the three age groups (*P <* 0.001)

***Significant difference in the three age groups (1.13 < 3.33 < 4.22, *P <* 0.001)

****Significant difference in the three age groups (0.22 < 0.48,0.56, *P <* 0.001)

*****Significant difference between boys and girls (*P <* 0.001)

******Significant difference between boys and girls (*P <* 0.001)

The prevalence of the primary and permanent teeth caries in the three age groups showed significant difference (*P <* 0.001). The prevalence of primary and permanent teeth caries among boys were 66.98 % and 22.95 % respectively, and 64.01 % and 33.74 % for girls. The prevalence of permanent teeth caries between boys and girls showed significant difference (*P <* 0.001). The dmft scores of the primary and permanent teeth in the three age groups showed significant difference (*P <* 0.001). The dmft scores of permanent teeth between boys and girls showed significant difference (*P <* 0.001).

### Caries experience (dmft) and oral health-related behaviours studied

Caries experience according to oral health-related behaviours studied is shown in Table 3. Higher primary teeth dmft scores were found in migrant children who performed tooth brushing less than twice daily, had initial brushing age >3 had sweet snacks before sleep without brushing teeth often or occasionally. Higher permanent teeth dmft scores were found in migrant children who had snacks daily.

**Table 3:** Caries experience (dmft) and oral health-related behaviours studied

Oral health-related behaviours (%, N)	Primary teeth dmft (SD)	Permanent teeth dmft (SD)
Tooth brushing twice or more daily (*N =* 1323)		
Yes (31.2 %, 413)	2.35 (2.93)*	0.41 (0.46)
No (68.8 %, 910)	2.91 (3.04)*	0.51 (0.60)
Age of initial brushing (*N =* 1170)		
≤ 3 (18.2 %, 213)	2.59 (2.90**	0.41 (0.75)
> 3 (81.8 %, 957)	3.10 (3.19)**	0.46 (0.87)
Sweet snacks before sleep without tooth brushing (*N =* 1323)		
Often/Occasionally (46.4 %,614)	2.96 (3.06)***	0.48 (0.86)
Never (53.6 %, 709)	2.54 (2.97)***	0.40 (0.81)
Daily sweet snacks (*N =* 1323)		
Yes (18.8 %, 249)	2.78 (2.96)	0.75 (1.04)****
No (81.2 %, 1074)	2.72 (3.03)	0.44 (0.77)****

*^,^ **^,^ ***Significant difference (*P <* 0.05)

****Significant difference (*P <* 0.001)

### Caries experience (dmft) and parents’ knowledge/attitudes of the child’s oral health

Caries experience according to parents’ knowledge/attitudes of the child’s oral health is shown in Table 4. The proportion of the surveyed parents who lacked knowledge about oral health was between 19.9 % and 68.8 %, and 2 % to 16.6 % of the respondents showed unscientific knowledge about oral health. However, neither primary nor permanent teeth dmft were significantly associated with parents’ knowledge/attitudes of child’s oral health.

**Table 4:** Caries experience (dmft) and parents’ knowledge/attitudes of the child’s oral health

Parents’ knowledge/attitude of the child’s oral health(%, N)	Primary teeth dmft (SD)	Permanent teeth dmft (SD)
Teeth are born good or bad, no correlation with the protection		
Agree (7.0 %, 92)	2.13 (2.65)	0.43 (0.79)
Disagree (64.7 %, 856)	2.67 (3.02)	0.46 (0.86)
Unknown (28.3 %, 375)	3.01 (3.07)	0.37 (0.80)
Oral health is important to life		
Agree (78.2 %, 1034)	2.70 (3.00)	0.47 (0.85)
Disagree (2.0 %, 26)	2.73 (3.42)	0.23 (0.65)
Unknown (19.9 %, 263)	2.90 (3.06)	0.33 (0.79)
It is important to protect the first permanent teeth of children		
Agree (47.2 %, 625)	2.69 (3.09)	0.47 (0.87)
Disagree (4.6 %, 61)	1.93 (2.54)	0.36 (0.78)
Unknown (48.2 %, 637)	2.85 (2.98)	0.41 (0.81)
No need to treat the bad primary teeth		
Agree (11.0 %, 145)	2.29 (2.74)	0.48 (0.86)
Disagree (52.5 %, 695)	2.69 (3.05)	0.48 (0.88)
Unknown (36.5 %, 483)	2.93 (3.04)	0.37 (0.75)
Pit and fissure sealant can prevent dental caries of children		
Agree (14.6 %, 193)	2.88 (3.20)	0.54 (0.97)
Disagree (16.6 %, 220)	2.40 (3.02)	0.50 (0.90)
Unknown (68.8 %, 910)	2.79 (2.97)	0.40 (0.78)
Tooth brushing twice a day can protect teeth		
Agree (70.4 %, 931)	2.74 (3.05)	0.47 (0.86)
Disagree (4.5 %, 59)	2.31 (2.43)	0.54 (1.01)
Unknown (25.2 %, 333)	2.80 (3.02)	0.33 (0.73)

### Dental visit

In the past 12 months, 272 (20.56 %) of the surveyed children went to the dental clinics and the average visiting time was 2.1 times with the median being 1.0. The main treatment for the most recent visiting was dental extraction (29.17 %), teeth examination (21.18 %) and dental trauma treatment (11.81 %). The visited dental institutions were mainly private clinics (22.43 %), community health service centres (22.06 %) and district hospitals (20.59 %). The main reasons for choosing these institutions were close proximity and convenience (26.25 %), qualified and professional medical techniques (23.75 %) and reliable doctors (18.44 %). The medical expenditures (including transportation fees) was 193.3 Yuan (30.68 $, exchange rates in January 1, 2012) on average with the median being 60.0 Yuan (9.52 $, exchange rates in January 1, 2012). During the treatment, the number of parents accompanying the children was 1.0 as the median and 9.56 % of the children had no parent there with them.

### Relationship between dmft scores and the selected independent variables

Multiple linear regression analysis showed that after the age factor was controlled, higher primary teeth dmft scores were found in migrant children who performed tooth brushing less than twice daily, had sweet snacks before sleep without brushing their teeth, and had a dental visit within the past 12 months. The multiple linear regression analysis also showed that after the age factor was controlled, the higher permanent teeth dmft scores were found among the girls, who had daily sweet snacks (Table 5).

**Table 5:** Multiple regression analysis for primary and permanent teeth dmft scores (*N =*1323)

Independent variables	Group	Beta	SE	95 % CI	*P*-value
Primary teeth					
Brushing twice or more daily	Yes	−0.610	0.188	−0.978–0.241	<0.05
	No^a^				
Sweet snacks before Sleep without tooth brushing	Often⁄Occasionally	0.407	0.178	0.056–0.757	<0.05
	Never^a^				
Dental visit within the past 12 months	Yes	1.042	0.214	0.622–1.462	<0.001
	No^a^				
Intercept		2.817	0.426		<0.001
Permanent teeth					
Gender	Boys	−0.182	0.049	−0.287–0.093	<0.001
	Girls^a^				
Daily sweet snacks	Yes	0.189	0.060	0.071–0.308	<0.05
	No^a^				
Intercept		0.518	0.125		<0.001

^a^Reference category, Primary teeth Adjusted R^2^ = 0.227, Permanent teeth Adjusted R^2^ = 0.069

## Discussion

Considering that Pudong New Area covers a vast territory and a geographically widespread distribution of migrant workers’ children, this study used a randomised multi-stage and cluster sampling method to include the children of migrant primary schools. In multi-stage cluster sampling, representative clusters were chosen and children within the chosen cluster were sampled. This is a cost-effective and efficient method of finding an adequate sample population. In this survey, we recruited a large sample size and a high response rate of migrant children; the support from the migrant school was the major reason for the satisfactory outcome.

Compared with the surveyed results of dental caries status of public primary school students [12], the prevalence of caries and dmft scores of the migrant students were higher. Similar findings have been published elsewhere. The 2003 United Kingdom Children’s Dental Health Survey [13] showed that children attending deprived primary schools were reported to have experienced more tooth decay than children in non-deprived schools. Another study, from Austria, has found that children whose parents were migrants were at greater risk of dental caries [14]. The migrant children had higher prevalence of caries and dmft scores were verified by many studies and reports [1, 15–17]. Having settled down in cities, the migrant children might have more delicious food, more opportunities for sweet snacks than before they lived in rural areas, so they have higher risk of dental caries [18, 19]. Besides, these children of migrant population had some bad oral health-related habits such as 68.8 % of surveyed migrant children brushing their teeth less than twice daily, 81.8 % of surveyed migrant children’ initial tooth brushing age >3 and 46.4 % of surveyed migrant children had sweet snacks before sleep without tooth brushing often or occasionally.

In terms of age, significant differences (*P <* 0.001) were found among migrant children in the prevalence of dental caries and dmft scores for primary and permanent dentition in this study. These findings are very similar to those outlined in previous studies [20–22]^.^ It is suggested that because primary migrant school children are in a period of mixed dentition and will gradually lose their primary teeth and develop permanent teeth over time, this leads to a reduction in the primary teeth caries status of 11–12 years old children compared with 7–8 years old children and a growth in the permanent teeth caries status of 11–12 years old children compared with 7–8 years old children. It was observed that girls had higher permanent teeth caries rate than had the boys. This finding also concurs with that of the previous surveys [10, 23].

The dental caries filling rate of children from migrant schools was also much lower than the indicator (31.2 % dental caries filling rate for public primary and middle school students of Shanghai) raised in 2008 [24], and still lagged behind the lowest target of the “Global Oral Health” of WHO (the filling rate in rural areas should be at least 15 %) [10]. The result might be related to the fact that migrant students without a local urban HuKou, unlike their counterparts in local public schools, were not entitled to oral-related public health services funded by the tax revenue such as dental caries filling, the pit and fissure sealant of the first permanent teeth. A national health insurance scheme subsidized by local governments targets those who own a local HuKou but are not employed by an organization, including full-time students, excluded migrant students. Besides, the income of migrant households was lower compared to their local counterparts. The aforementioned reasons reduced the probability of receiving hospital-based treatment services among migrant students. The low education level of parents and the language barrier (the migrant population have different dialects with various accents because they come from different provinces) may be another reason for lack of easy access to dental caries care.

The children of migrant schools in this study, however, experienced fewer primary tooth caries when they practiced twice or more tooth brushing daily. This finding is in agreement with a number of earlier studies in various child populations [25–27]. High primary and permanent tooth caries experience was found in migrant children who consumed snacks before sleep without brushing their teeth and sweet snacks daily respectively. Previous studies have shown that children with bedtime eating habits have high risk. Obviously, this habit provides two critical caries-promoting conditions: a large volume of substrates (sucrose, glucose and fructose) and sufficient time for bacteria to produce the acid products. While the accumulation of the acid products damages the teeth directly, it also facilitates the adhesion of bacteria on the teeth surface and the interaction between bacteria. In addition, owing to the change of the permeability of bacterial plaque, the buffering capacity of saliva is weakened or diminished. Ultimately, a prolonged exposure of the teeth in the acidic environment causes the teeth etching and caries [28–30].

A higher caries experience was found in the children who visited a dentist. This finding was also found in a previous study [31, 32]. Problem-oriented dental care seeking behaviour is common in China. It is plausible that children were brought to visit dentists for pain and infection. A previous study investigated the categories of oral health services that migrant students sought during their visits to outpatient departments of stomatology in the past year and found that the most common service received was dental extraction, taking up 29.17 percent of all the categories. Efforts should be made to promote primary dental care. Community oral-health programmes can be designed and implemented to ensure that those needs regarding dental caries are met [27].

Compared with the results of the third national oral-health survey [10], parents’ knowledge/attitudes of children’s oral health in our study was more satisfactory. This might be correlated with the rapid economic growth in the last 10 years, the comprehensive dissemination of television, internet and other media which made it easier for the parents to access the information of children’s oral health. However, there exists a remarkable proportion of the surveyed parents who lacked knowledge about children’s oral health, and some parents even held unscientific perspectives towards oral health, which could be attributed to their low socioeconomic status and other living necessities which were regarded as more important than the oral health of children [33]. The involvement of local communities could be encouraged to promote targeted prevention measures to improve the knowledge-attitude-behaviour of the migrant population towards oral health in order to improve the child’ caries status and oral health-related behaviours.

## Limitations of the study

In this study, we assessed the dental caries status of the children from migrant primary schools in Shanghai Pudong New Area. However, there are a few weaknesses in this study. Firstly, the surveyed children selected using the multi-stage cluster sampling method may not be as representative as those selected through random sampling. Secondly, attending migrant primary school is not compulsory for migrant children. Some children of the migrant population might not go to migrant schools and were not able to be included in this survey. Moreover, those who did not attend on the day of caries examination, without an informed consent or did not submit the questionnaire were also excluded. Although the number should be small, it could affect the prevalence and caries experience of children of migrant primary schools. Thirdly, the sample size was calculated based on the results of the third national oral-health survey conducted in 2005, which may be not adequate for this study. Finally, we were unable to ascertain the level of parental bias in responding to self-reported questions.

## Conclusions

In this survey, students from migrant primary schools in Shanghai Pudong New Area had bad conditions of dental caries and most of the carious teeth were left untreated. The caries experience was associated with tooth brushing habits, snacking habits, dental visits and gender.

### Availability of supporting data

The dataset supporting the conclusions of this article is included within its additional file.

## Abbreviations

WHO: world health organization
SD: standard deviation
ANOVA: analysis of variance

## References

[1] Abdul-Razak B, Chrysoula O, Martin JK (2007). Dental health, received care, and treatment needs in 11- to 13-year-old children with immigrant background in Heidelberg, Germany. Int J Paediatr Dent.

[2] Hu X, Cook S, Salazar MA (2008). Internal migration and health in China. Lancet.

[3] Net CGA (2013). China floating population development report in.

[4] National Bureau of Statistics of China (2012). Tabulation on the 2010 population census of People’s Republic of China (Book I, Book II, Book III).

[5] Li X, Zhang L, Fang X (2010). Schooling of migrant children in China: Perspectives of school teachers. Vulnerable Children and Youth Studies.

[6] Yuan, X, Fang, X, Liu, et al. Educational settings and city adaptation of migrant children. Journal of Beijing Normal University (Social Science), 2009, 5:25–32

[7] Chen X, Wang L, Wang Z (2007). Shyness-sensitivity and social, school, and psychological adjustment in rural migrant and urban children in China. Child Dev.

[8] 2013 Shanghai National Economy and Social Development Statistical Bulletin. http://www.stats-sh.gov.cn/sjfb/201402/267416.html. [Accessed on November 25, 2014].

[9] Population and Family Planning Management Center of Pudong New Area. Data analysis on population of Pudong New Area of Shanghai in 2013. Available at: http://rkjs.pudong.gov.cn/Info_Content.aspx?Item_Id=1820. [Accessed on November 26, 2014]

[10] Qi XQ (2008). Report of the third national survey of oral health.

[11] World Health Organization (1997). Oral Health Surveys – Basic Methods. 4th version.

[12] Qi H, Gu MR, Hu J (2009). Investigation and analysis of caries among school students in Pudong New Area[J] (in Chinese). Modern Preventive Medicine.

[13] United Kingdom National Technical Reports (2003). Children’s Dental Health in the UK, National Statistics.

[14] Cvikl B, Haubenberger-Praml G, Drabo P (2014). Migration background is associated with caries in Viennese school children, even if parents have received a higher education. BMC Oral Health.

[15] Gao XL, Colman M, Lin HC (2011). Oral health status of rural–urban migrant children in South China[J]. Int J Paediatr Dent.

[16] Kühnisch J, Senkel H, Heinrich-Weltzien R (2003). Comparative study on the dental health of German and immigrant 8- to 10-years olds in the Westphalian Ennepe-Ruhr district (English Summary). Gesundheits-wesen.

[17] Van Steenkiste M (2002). Caries preventive strategies in the light of the actual caries decline- An analysis from a public health point of view (English Summary). Oralprophylaxe.

[18] Rasmia H, Paula W, Paula M, Simon K, Anne M (2012). Dental caries and its association with diet and dental erosion in Libyan schoolchildren. Int J Paediatr Dent.

[19] Li Y, Zhang Y, Yang R (2011). Associations of social and behavioral factors with early childhood caries in Xiamen city in China. Int J Paediatr Dent.

[20] Cheng YC, Huang HK, Wu CH (2014). Correlation between dental caries and diet, oral hygiene habits, and other indicators among elementary school students in Xiulin Township, Hualien County, Taiwan[J]. Tzu Chi Medical Journal.

[21] Yu HJ, Huang ST, Chen HS (2008). Association of dietary and dental hygiene habits with the prevalence of dental caries of 6–12 year-old schoolchildren in eastern Taiwan[J]. Taiwan J Oral Med Sci.

[22] Yang YH, Hu SW, Shieh TY (2006). Elementary Schoolchildren’s Nutrition and Health Survey in Taiwan 2001-2002-an association of the caries condition with the consumption of sweet snacks and dairy products [J]. Chin Dent J.

[23] Zhang S, Liu J, Lo EC (2014). Dental and periodontal status of 12-year-old Bulang children in China.

[24] Women and Children Working Committee of Shanghai. Main indicator realization status of the Eleventh Five-year Plan of Children Development of Shanghai in 2008. Available at: http://www.few.gov.cn/portal/html/dbqk/guihuadabiaoqingkuang/ertongzhuyaomubiao/2009/1119/15060.html. [Accessed on December 10, 2014]

[25] Chu CH, Wong AW, Lo EC (2008). Oral health status and behaviours of children in rural districts of Cambodia. Int Dental J.

[26] Harris R, Nicoll AD, Adair PM, Pine CM (2004). Risk factors for dental caries in young children: a systematic review of the literature. Community Dent Health.

[27] World Health Organization: The Liverpool declaration: promoting oral health in the 21st century. http://www.who.int/oral_health/events/liverpool_declaration/en/ [Accessed on 10 May 2015].

[28] Vadiakas G (2008). Case definition, aetiology and risk assessment of early childhood caries (ECC): a revisited review. Eur Arch Paediatr Dent.

[29] AI Majed I (2002). Risk factors for dental erosion in 5–6 year old and 12–14 year old boys in Saudi Arabia. Community Dent Oral Epidemiol.

[30] Zero DT (2004). Sugars – the arch criminal?. Caries Res.

[31] Chu CH, Ho PL, Lo EC (2012). Oral health status and behaviours of preschool children in Hong Kong. BMC Public Health.

[32] Zhang S, Liu J, Lo EC (2013). Dental caries status of Dai preschool children in Yunnan Province China. BMC Oral Health.

[33] Wong FKD (2008). Rural migrant works in urban China: living a marginalized life. Int J Soc Welf.

